# Microwell Plate-Based Dynamic Light Scattering as a High-Throughput Characterization Tool in Biopharmaceutical Development

**DOI:** 10.3390/pharmaceutics13020172

**Published:** 2021-01-27

**Authors:** Katharina Dauer, Stefania Pfeiffer-Marek, Walter Kamm, Karl G. Wagner

**Affiliations:** 1 Department of Pharmaceutical Technology and Biopharmaceutics, University of Bonn, 53121 Bonn, Germany; katharina.dauer@uni-bonn.de; 2 Tides Drug Product Pre-Development Sciences, Sanofi-Aventis Deutschland GmbH, Industrial Park Hoechst, 65926 Frankfurt am Main, Germany; stefania.pfeiffer-marek@sanofi.com (S.P.-M.); walter.kamm@sanofi.com (W.K.)

**Keywords:** high-throughput technologies, dynamic light scattering, variability, diffusion interaction parameter, protein-solvent interaction, protein characterization, formulation development, biopharmaceuticals

## Abstract

High-throughput light scattering instruments are widely used in screening of biopharmaceutical formulations and can be easily incorporated into processes by utilizing multi-well plate formats. High-throughput plate readers are helpful tools to assess the aggregation tendency and colloidal stability of biological drug candidates based on the diffusion self-interaction parameter (*k_D_*). However, plate readers evoke issues about the precision and variability of determined data. In this article, we report about the statistical evaluation of intra- and inter-plate variability (384-well plates) for the *k_D_* analysis of protein and peptide solutions. ANOVA revealed no significant differences between the runs. In conclusion, the reliability and precision of *k_D_* was dependent on the plate position of the sample replicates and *k_D_* value. Positive *k_D_* values (57.0 mL/g, coefficients of variation (*CV*) 8.9%) showed a lower variability compared to negative *k_D_* values (−14.8 mL/g, *CV* 13.4%). The variability of *k_D_* was not reduced using more data points (120 vs. 30). A *k_D_* analysis exclusively based on center wells showed a lower *CV* (<2%) compared to edge wells (5–12%) or a combination of edge and center wells (2–5%). We present plate designs for *k_D_* analysis within the early formulation development, screening up to 20 formulations consuming less than 50 mg of active pharmaceutical ingredient (API).

## 1. Introduction

Biopharmaceuticals play an increasingly important role in the treatment of various diseases, and hundreds of these biopharmaceuticals are in the clinical development pipeline [1]. The development of stable liquid protein formulations is often challenging and time- and material-consuming [2]. High-throughput (HT) approaches are often used to analyze multiple formulations rapidly and facilitate the selection for successful therapeutics [3]. However, the most challenging task within the early development of protein-based therapeutics is their limited stability due to a labile nature of structural integrity. This aspect evokes a high susceptibility to physical and chemical degradation at any point throughout the lifecycle of a protein and even during administration of its liquid solution [4]. In this context, protein aggregation is one of the most common physical instabilities. The tendency to aggregate depends on intermolecular interactions, and formed aggregates can considerably range in size, up to visible particles, endangering the safety and efficacy of the drug product due to an increased immunogenicity [5]. Colloidal stability results from protein–protein interactions and is also an important factor for protein aggregation [2]. Before biopharmaceuticals enter preclinical development, the aggregation tendency is an essential criteria for selecting successful drug candidates [4]. The propensity of proteins for self-association and aggregation is mostly evaluated by analytical approaches commonly based on light scattering [6]. Dynamic light scattering (DLS) enables the determination of the diffusion coefficient (*D*), particle size, and particle size distribution. Concentration-dependent changes of the diffusion coefficient determine the diffusion self-interaction parameter (*k_D_*) and are indicative for colloidal stability, which, in return, helps to estimate the aggregation tendency [2]. HT and low-volume methods are highly preferred to estimate the protein stability, solubility, and viscosity, avoiding the use of time- and API (active pharmaceutical ingredient)-consuming studies [7,8]. HT methods can be easily incorporated into processes by utilizing multi-well plate formats (e.g., “plate readers”). Microtiter plates are containers with a grid of small, open wells (e.g., six- up to 1536-wells per plate) [9]. HT instruments based on light scattering are widely used in research and development [10] and have been developed especially for the accelerated screening and characterization of drug candidates, with a focus on evaluating the protein stability [2]. The most commonly used plates for light scattering experiments are polystyrene (PS) or polyvinyl-chloride plates. PS is frequently used as the material of choice, since it shows a limited tendency for protein binding [11,12,13]. The available amount of API is typically limited in the early phase of development; thus, higher density microplates (384- or 1536-wells) are preferred for screening [7]. The determination of *k_D_* using HT-plate readers raises questions about the precision and variability of the obtained data. Intra- and inter-assay coefficients of variation (*CV*) are used to express the precision of the analytical results [14]. A low *CV* between sample replicates is crucial in demonstrating the assay was well-run, whereas a high *CV* can highlight inconsistencies among sample replicates in data interpretation. An intra-assay *CV* defines the variation between data points within an assay in the same run, whereas an inter-assay *CV* is the variation between runs of sample replicates on different plates [15]. Experimental results with a poor intra- or inter-assay *CV* > 10% commonly represent a poor pipetting technique [16] or a mishandling due to a high viscosity of the pipetted samples. Foam or air bubbles on the sample surface generated during liquid pipetting can scatter light and interfere with accurate plate reading [17,18]. Furthermore, the splashing of samples between wells [19], drying out of wells (evaporation) [13,20], or inhomogeneous samples can also play a major role [16]. In addition, well-to-well variations on individual plates can result in varying analytical values (e.g., “edge effects”) [11,20]. Plate variability was investigated for a large number of immunological and biochemical assays (e.g., ELISA) [12,14,21,22,23]. However, the main focus was hardly ever put on the evaluation of plate variabilities for HT formulation screening purposes (e.g., protein–protein or protein–excipient interactions). HT screening certainly allows the rapid screening of diverse formulations but leads also to a multiplication of the data generated. Consequently, it is challenging to reliably identify systematic errors and make a decision for the best biophysical properties from a set of multiple formulations [2]. Shi et al. [24] focused on a statistical evaluation of the plate-based *k_D_* method with 96-well plates studying the weak protein–protein interactions of lysozyme. They reported a location variability on the 96-well plates of >25% and an inter-day variability of >10%. However, variability assessments, particularly for 384-well plates, and the extent of the variation achieving the considered requirements during early development were not extensively studied until now. In this study, a *k_D_* method based on a DLS plate reader was qualified with respect to these caveats. The first variability study was designed with the model protein lysozyme in acetate buffer, pH 4.5, in the absence (positive *k_D_*) or presence of 400-mM sodium chloride (negative *k_D_*). Based on these findings, the second variability study was performed with a model peptide drug (peptide-12) and applied to the HT formulation screening. Peptide-12 was formulated in acetate, pH 4.5 (positive *k_D_*), and phosphate, pH 6.0 buffer (negative *k_D_*), upon addition of an ionic (NaCl) and a nonionic (glycerol) tonicity agent. Due to multiple factors that may affect data collection and interpretation, the samples were positioned on 384-well plates. Various plate designs were tested, with the aim of detecting patterns, such as edge effects or trends along rows or columns. The evaluation was performed by analysis of variance (ANOVA) and included intra- and inter-plate variance analyses. Based on the lysozyme, we also investigated the need of an adequate outlier test and the number of minimum concentration steps for a reliable *k_D_* analysis. Finally, we recommend two plate designs for HT formulation screening based on the identified systematic errors, providing appropriate and high-quality experimental data collected with a low amount of API.

## 2. Materials and Methods

### 2.1. Materials

Lysozyme from chicken egg whites, lyophilized powder (Cat. No. L6876), acetic acid, ortho-phosphoric acid 85%, trifluoroacetic acid (TFA), acetonitrile, and DMSO were obtained from Merck Chemicals GmbH (Darmstadt, Germany). Synthetic peptide-12 [25,26] (4.7 kDa, purity 97.5%, isoelectric point (pI) 6.7) as a freeze-dried powder was kindly provided by Sanofi-Aventis Deutschland GmbH (Frankfurt, Germany). Sodium chloride, sodium acetate trihydrate, and glycerol 85% were purchased from Aug. Hedinger GmbH & Co. KG (Stuttgart, Germany). All chemicals were of analytical grade or equivalent purity. Milli-Q^®^ water was used for the preparation of all aqueous solutions (obtained through a Milli-Q^®^ Millipore filter system, Millipore Co., Bedford, MA, USA).

### 2.2. Sample Preparation of Lysozyme Formulations

Two different batches of lysozyme (LysSig2243 and LysSig5161) were dissolved in 20-mM acetate buffer, pH 4.5, and then extensively dialyzed into the same buffer using 3.5-kDa MWCO dialysis cassettes (Slide-A-Lyzer^TM^ 30 mL, 66,230, Pierce Biotechnology, Rockford, IL, USA). The dialyzed lysozyme stock solutions were diluted to 17 mg/mL. Afterwards, all solutions for sample preparations were multistep filtered under laminar flow through 0.22-μm, 0.1-µm, and 0.02-µm sterile filters (0.22 μm and 0.1 μm, 33 mm, Low Protein-Binding Durapore^®^ (PVDF) Membrane, Millex^®^ GV and Millex^®^ VV, Sterile R, Merck Millipore Ltd., Tullagreen, Ireland; 0.02 μm, 25 mm, Inorganic Membrane Filter Anotop^TM^ 25, GE Healthcare UK Limited, Whatman, Germany). Series of dilutions was used to prepare the concentration series (2–14-mg/mL lysozyme) in the absence or presence of 400-mM sodium chloride (NaCl). Formulation placebos were also prepared containing all excipients except lysozyme.

### 2.3. High-Throughput Preparation of Peptide-12 Formulations

Peptide-12 formulations were prepared by a high-throughput robotic system called FRED (Formulation Robot for Early Projects and Development). Stock solutions of the formulation components (peptide-12, acetate buffer, pH 4.5, phosphate buffer, pH 6.0, glycerol, and NaCl) were sterile-filtered under laminar flow (Herasafe KS, Fisher Scientific GmbH, Schwerte, Germany) with 0.1-μm PVDF filters (0.1 μm, 33 mm, Low Protein-Binding Durapore^®^ (PVDF) Membrane, Millex VV, Merck Millipore Ltd., Tullagreen, Ireland). The FRED equipped with a LISSY robot (Zinsser Analytic GmbH, Eschborn, Germany) and weighing platform (Unchained Labs Junior with powder dispensing unit, Unchained Labs, Pleasanton, CA, USA) combined the appropriate volumes of the n-fold concentrated stock solutions (Automation Tips for Qiagen™ Workstations, 1100-µL polypropylene tips, Thermo Fisher Scientific GmbH, Dreieich, Germany) to generate the formulations. All components were combined in sterile vessels containing a sterilized magnetic stirring bar (Teflon^®^ PTFE-encapsulated, Spinbar^®^ micro, VWR Chemicals, Leuven, Belgium). The exact weights of the added stock solutions were recorded after each pipetting step by the robotic weighing platform. The final formulations were homogenized by stirring at 200 rpm for 15 min.

### 2.4. Determination of Protein and Peptide Concentration

Concentration was determined using a NanoDrop^TM^ One spectrophotometer (ThermoScientific, Waltham, WA, USA). Aliquots (3 μL, n = 6) of each sample were measured at 280 nm, and concentrations were calculated using absorbance and the mass extinction coefficient for lysozyme (*E_1%_* = 2.64 mL·mg^−1^·cm^−1^) and the molar extinction coefficient for peptide-12 (*E_M_* = 5690 M^−1^·cm^−1^). The deviation between the 6 replicates was <2%, whereas the deviations of the lysozyme and peptide-12 concentrations were <4% and <5% on average, respectively, related to the theoretical concentrations.

### 2.5. Determination of Denaturation Temperature (T_m_) by Intrinsic Fluorescence Spectroscopy (IFS)

*T_m_* of lysozyme in solution was investigated using a UNit instrument (Unchained Labs, Pleasanton, CA, USA). Each sample (8.65 μL) was loaded into microcuvette arrays (UNi) with the Optim^®^II device (Avacta Analytical, Wetherby, UK) and subjected to a linear thermal ramp from 25 °C to 95 °C at a heating rate of 0.5 °C/min. The samples were analyzed in triplicate for intrinsic fluorescence evoked by the intrinsic tryptophan residues and static light scattering (SLS) at 266 nm. The intrinsic fluorescence data were analyzed using the barycentric mean (BCM). BCM was plotted against temperature for each of the lysozyme samples tested. Data were analyzed using the UNit Analysis software v2.1 (Unchained Labs, Pleasanton, CA, USA). Curves of sigmoidal shape and the inflection point of the fit were assigned as denaturation temperature *T_m_*.

### 2.6. Molecular Weight (M_w_) and Purity of Lysozyme by UHPLC-RP/UV-MS

Lysozyme was solubilized in DMSO at a concentration of 1 mg/mL for 1 h applying gentle agitation. Afterwards, the solutions were centrifuged for 20 min at 3000 RCF (Centrifuge 5810, Rotor A-2-DWP, Eppendorf AG, Hamburg, Germany). Three-microliter aliquots of the supernatant were injected on an UHPLC (ultra high performance liquid chromatography) system consisting of a Waters Acquity I-class LC equipped with a photodiode-array detector (PDA) that was hyphenated to a Waters LCT Premier TOF (Waters GmbH, Eschborn, Germany). Sample separation was accomplished on a C18 column (Waters Acquity CSH C18, 1.7 µm, 2.1 × 150 mm) operated at 50 °C, and UV absorption was detected at 214 nm. Solvent A consisted of water with 500-ppm TFA and solvent B composed of acetonitrile with 450-ppm TFA running with a constant flow of 0.5 mL/min. After a column load phase, the gradient increases the amount of acetonitrile from 20 to 75% in a linear curve during 23 min, followed by a wash phase. The mass spectrometer, equipped with an electrospray ionization (ESI) source, was operated according to the manufacturer’s instructions in the W flight mode. Spectra were recorded with positive polarity in the range of 300 to 3000 m/z at a resolution of about 10,000. By analyzing the MS (mass spectroscopy) spectra of the main peak, the expected mass and isotopic distribution of each batch was confirmed. Lysozyme purity was calculated by the following equation after manual integration of the UV chromatogram:

(1) $$Purity\;\left(\%\right)=\frac{peak\;area_{lysozyme}}{total\;peak\;area}\cdot 100\%$$

where *peak area_lysozyme_* is the area of the chromatographic peak corresponding to the product, and total peak area is the sum of the areas of all the peaks present in the chromatogram.

### 2.7. Surface Tension Measurements by Wilhelmy Plate Method

Surface tension of lysozyme solutions in 20-mM acetate buffer, pH 4.5, was measured using a K100 tensiometer (Krüss GmbH, Hamburg, Germany) after an equilibrating time of 10 min at 25.0 ± 0.5 °C. The surface tension (air–water) was measured using the Wilhelmy plate method (i.e., force balance method) [27]. Experiments on each concentration were repeated four times.

### 2.8. Dynamic Viscosity Measurements

Dynamic viscosity of lysozyme solutions in 20-mM acetate buffer, pH 4.5, was measured using the microfluidic rheometer m-VROC^TM^ (RheoSense Inc., San Ramon, CA, USA) at 25.0 ± 0.5 °C. 600 µL of the sample were loaded into a 0.5-mL glass syringe (Hamilton Company, Reno, NV, USA). The syringe was then inserted into the rheometer. The system was rinsed by an initial injection of 100 µL of the sample. Measurements of each sample were repeated three times (160 µL per measurement).

### 2.9. Plate-Based DLS Measurement of the Diffusion Self-Interaction Parameter (k_D_)

*k_D_* values of formulations were determined by dynamic light scattering using a Wyatt DynaPro^®^ Plate Reader II (Wyatt Technology Europe GmbH, Dernbach, Germany). Lysozyme concentrations ranged from 2 to 14 mg/mL (8 steps) and peptide-12 concentrations from 2 to 10 mg/mL (5 steps). 30 µL-aliquots of each dilution were pipetted into 384-well microtiter plates (clear bottom, low volume, PS, Corning 3540, Corning GmbH HQ, Wiesbaden, Germany). Afterwards, the plates were centrifuged (Centrifuge 5430 R, Eppendorf AG, Hamburg, Germany) for 2 min at 600 RCF. One measurement included 15 acquisitions of 15 s at 25 °C (auto-attenuation mode). Data were collected with DYNAMICS^®^ software V7.8 (Wyatt Technology Corporation, Santa Barbara, CA, USA). Hydrodynamic radii (*R_H_*) and averaged intensities of particle sizes were calculated from the autocorrelation function (ACF) (regularization analysis method). ACFs are shown exemplarily in the Supplementary Materials, Tables S1 and S2. *k_D_* was manually calculated by a linear fit of the measured DLS-based diffusion coefficients (*D*) of each dilution series as a function of the protein or peptide concentration *c*; *D_0_* is the diffusion coefficient at an infinite dilution [28]:

(2) $$D\;=\;D_{0}\;\cdot \;\left(1\;+\;k_{D}\;\cdot \;c\right)$$

### 2.10. Variability and Statistics of Plate-Based DLS Measurements

Variance analysis was based on data collected by plate-based DLS measurements and included: (i) intra- and (ii) inter-plate variance analyses of lysozyme and peptide-12 formulations. “Run” was defined as each plate that was measured in a separate DLS experiment. Plates were measured immediately after pipetting and centrifugation. Animal-derived lysozyme extraction and lyophilization can induce significant batch-to-batch variations. Prior to statistical evaluation of the lysozyme formulations, batches (LysSig2243 and LysSig5161) were investigated in terms of their batch-to-batch consistency (*R_H(c__→0)_*, *k_D_*, *T_m_*, solution pH, *M_w_*, and purity). Grubbs’ test (extreme studentized deviate test) was used to identify outliers in the DLS raw data sets (diffusion coefficients for *k_D_* analysis) under the hypothesis that there were no outliers or there was one outlier in the dataset (*α* = 0.05). Datasets were previously tested for normality by the Kolmogorov-Smirnov test, and equality of variances was determined by *F*-tests (*α* = 0.05). The term “based on averaged data” means that diffusion coefficients to calculate *k_D_* were submitted to a Grubbs’ outlier analysis. Detected outliers were then excluded and not used for the calculation of *k_D_*. However, outliers were not excluded for *k_D_* determination within the dataset “based on raw data”, since the purpose was to evaluate the need of an appropriate outlier analysis.

**Figure 1:**
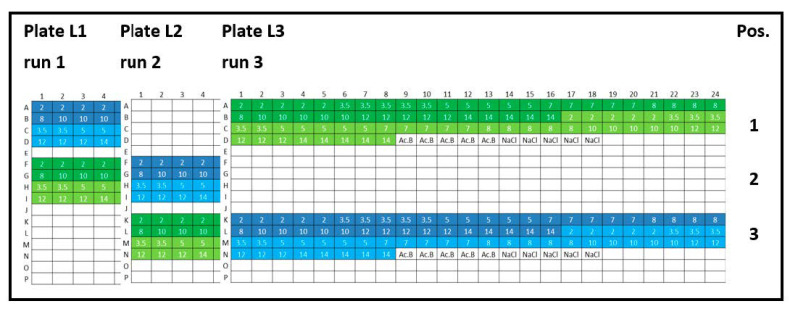
Experimental plate designs for intra-plate variability. Lysozyme (L): three 384-well plates were analyzed (run 1, run 2, and run 3), and the concentration series was arranged horizontally (2, 3.5, 5, 7, 8, 10, 12, and 14 mg/mL). Lysozyme batch types: LysSig2243 (blue), +400-mM NaCl (cyan) and LysSig5161 (green), +400-mM NaCl (light green). Control samples: Ac.B: 20-mM acetate buffer, pH 4.5, and NaCl: 400-mM of sodium chloride in acetate buffer, pH 4.5.

**Figure 2:**
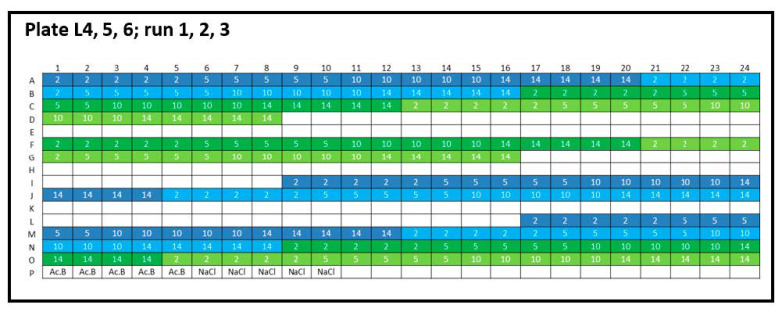
Experimental plate design for inter-plate variability. Lysozyme (L): three 384-well plates were analyzed (run 1, run 2, and run 3), and the concentration series was arranged horizontally (2, 5, 10, and 14 mg/mL). Lysozyme batch types: LysSig2243 (blue), +400-mM NaCl (cyan) and LysSig5161 (green), +400-mM NaCl (light green). Control samples were placed in row P: Ac.B: 20-mM acetate buffer, pH 4.5, and NaCl: 400-mM of sodium chloride in acetate buffer, pH 4.5.

**Figure A1:**
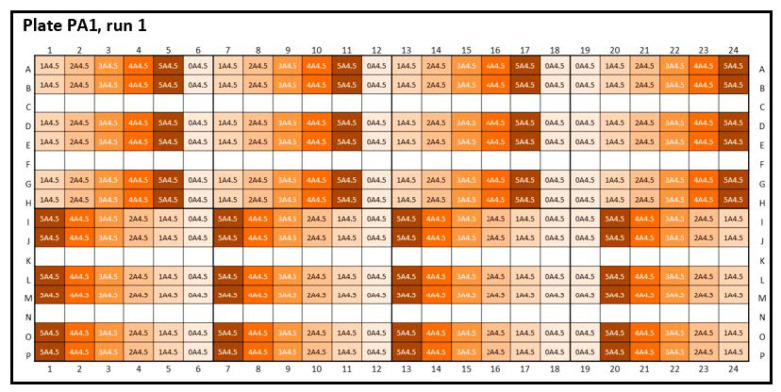
Experimental plate designs for intra-plate variability. Peptide-12 (P): two 384-well plates were analyzed: PA1 (positive *k_D_*): A4.5 = acetate buffer, pH 4.5, and the concentration series were arranged horizontally; ascending concentrations are placed on the upper half of the plate and descending concentrations on the bottom half (1 = 2 mg/mL, 2 = 4 mg/mL, 3 = 6 mg/mL, 4 = 8 mg/mL, 5 = 10 mg/mL API, and 0 = placebo). The design of plate PP1 (negative *k_D_*) for phosphate buffer, pH 6.0, was equal to plate PA1.

**Figure A2:**
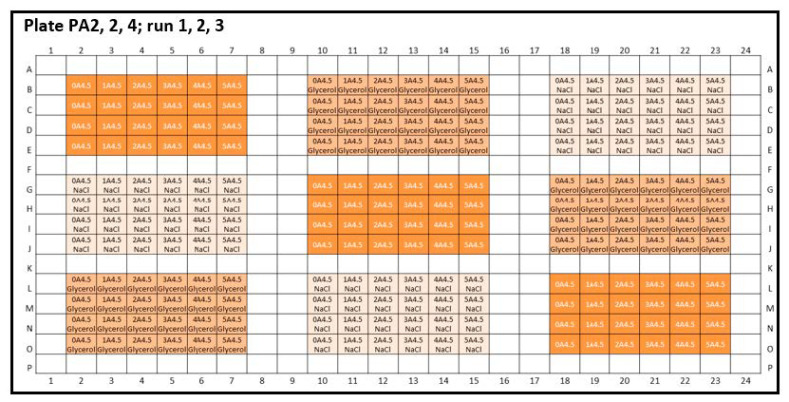
Experimental plate designs for inter-plate variability. Peptide-12 (P): three 384-well plates were analyzed: PA2-4: A4.5 = acetate buffer, pH 4.5; the concentration series were arranged horizontally; 4 replicates were positioned vertically. (1 = 2 mg/mL, 2 = 4 mg/mL, 3 = 6 mg/mL, 4 = 8 mg/mL, 5 = 10 mg/mL API, and 0 = placebo). Glycerol (orange) and sodium chloride (light orange) were added as tonicity agents, and formulations without tonicity agents were marked in dark orange.

(i) Intra-plate variability was calculated as the *CV* for lysozyme and peptide-12 formulations from different locations on 384-well plates (Figure 1, plate designs concerning peptide-12 and Appendix A, Figure A1). Intra-plate *CV* was calculated as the ratio of pooled *SD* from all samples and the overall mean. The average intra-plate *CV* was obtained by averaging the individual *CV* from each run. The null hypothesis was defined as: There is no difference between the plate positions 1, 2, and 3.

(ii) Inter-plate variability was calculated as the *CV* of the lysozyme and peptide-12 formulations measured at three consecutive runs but using identical plate designs (Figure 2, plate designs concerning peptide-12 and Appendix A, Figure A2). The null hypothesis was defined as: There is no difference between the runs 1, 2, and 3. Statistical evaluation of *D* and *k_D_* obtained from different formulations was performed by analysis of variance (one-way and repeated measures ANOVA, Microsoft Excel^®^ 2010, Microsoft Corporation, Redmond, WA, USA). If *F* > *F_crit_* and *p* ≤ 0.05, the null hypothesis was rejected, concluding that the difference between the population means was statistically significant. Statistical significance was depicted by asterisks (*) for *D* and *k_D_*. If necessary, post-hoc-corrected *t*-tests (two-samples assuming equal variances, Microsoft Excel^®^ 2010, Microsoft Corporation, Redmond, WA, USA) were applied in order to identify differences between tested groups.

## 3. Results and Discussion

We applied a HT light scattering method with 384-well plates for the investigation of the statistical variability between the positions (intra-plate variability), as well as to detect patterns, such as edge effects or trends along rows or columns, between runs (inter-plate variability).

**Figure 3:**
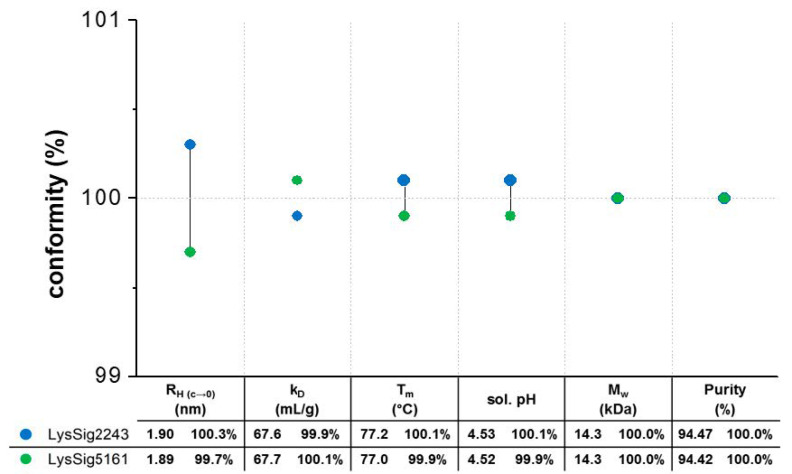
Batch conformity profile to evaluate the batch-to-batch variations: two lysozyme batches (blue spheres: LysSig2243 and green spheres: LysSig5161) were investigated in terms of their hydrodynamic (dynamic light scattering (DLS): hydrodynamic radii (*R_H(c__→0)_*) and diffusion self-interaction parameter (*k_D_*)), thermodynamic (intrinsic fluorescence spectroscopy (IFS): denaturation temperature (*T_m_*)), and physicochemical properties (solution pH and LC-MS: molecular weight (*M_w_)* and purity). The percentage conformity calculations were based on the averaged values of both batches.

Prior to the statistical evaluation, the lysozyme batches were investigated for their batch conformity. Accordingly, both batches showed equal hydrodynamic, thermodynamic, and physicochemical properties, resulting in identical molecular weights and equal denaturation temperatures (*T_m_*), solution pH values, and purities (Figure 3). The ACFs were smooth exponentials with a baseline of ~1.00 and low SOS (sum of squares) values (<20) (Supplementary Materials, Tables S1 and S2), demonstrating monomodal size distributions with low polydispersity and good-quality decays during the DLS experiments. The single-particle hydrodynamic radii (*R_H(c→0)_*) by DLS were determined by extrapolating the *R_H_* to an infinite dilution (c → 0) using lysozyme concentrations ranging from 2–7 mg/mL. The single-particle *R_H(c→0)_* for the lysozyme monomer in 20-mM acetate buffer at pH 4.5 was determined as 1.9 nm. In the presence of 400-mM NaCl, the *R_H(c→0)_* was 2.0 nm [29,30,31]. The *k_D_* values were calculated from *D* obtained from four concentrations (2, 5, 10, and 14 mg/mL lysozyme), three runs, five replicates per run, and three plate positions (total of 9 *k_D_* for each lysozyme batch). The ANOVA also showed no significant differences (*p* = 0.226) between the batches.

### 3.1. Variability and Statistics of Plate-Based DLS Measurements

In this study, we focused on the Grubbs’ test, as it is commonly used and recommended by the International Organization for Standardization (ISO), as well as being robust, reliable, and computationally simple [32]. The Grubbs’ test is a simple statistical test for outliers on correlation data in order to make a decision about the presence of a single outlier or the absence of outliers in a univariate dataset.

**Figure 4:**
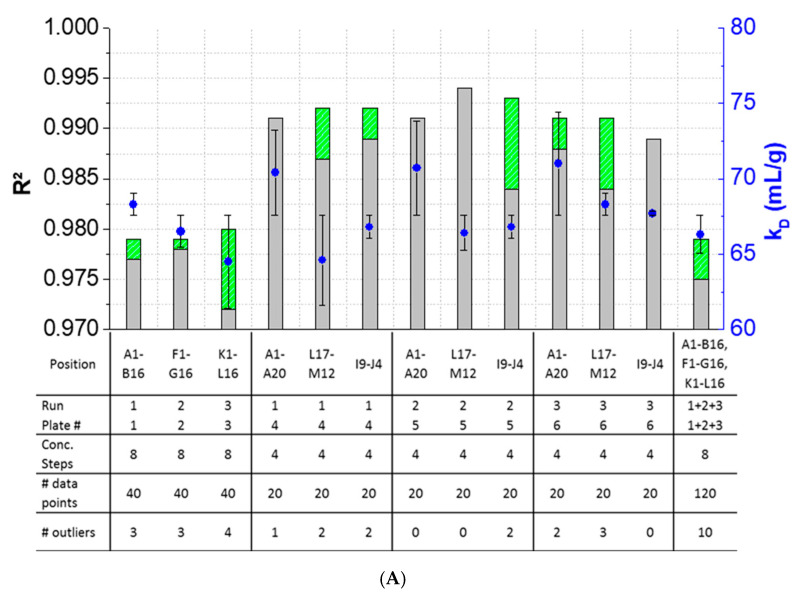

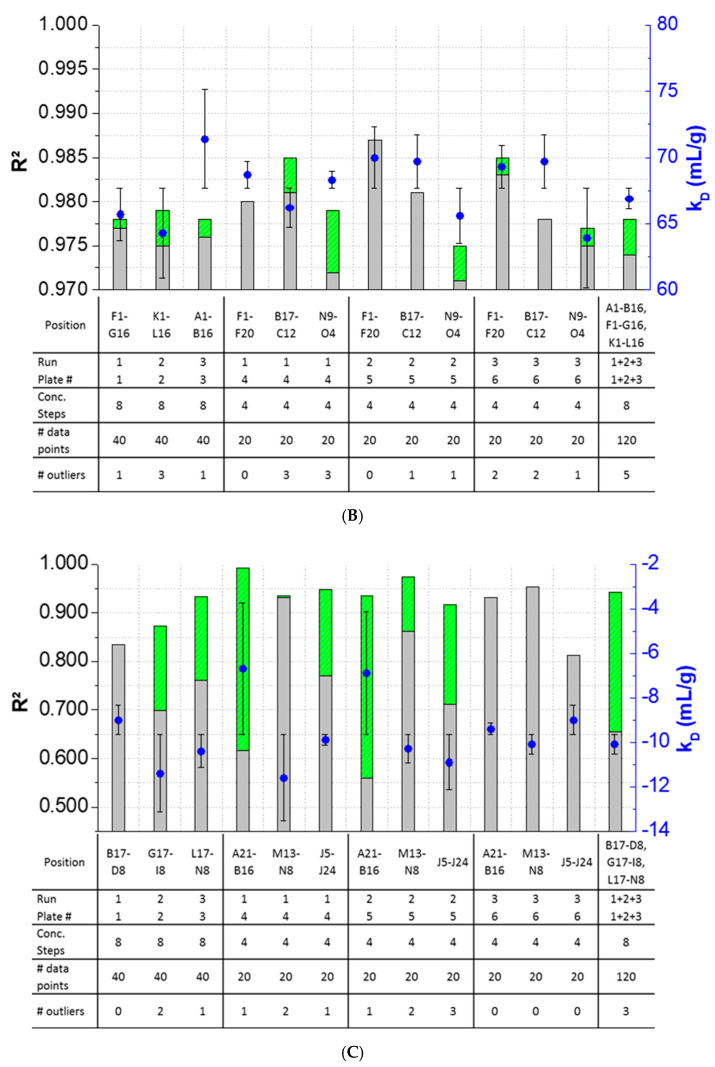

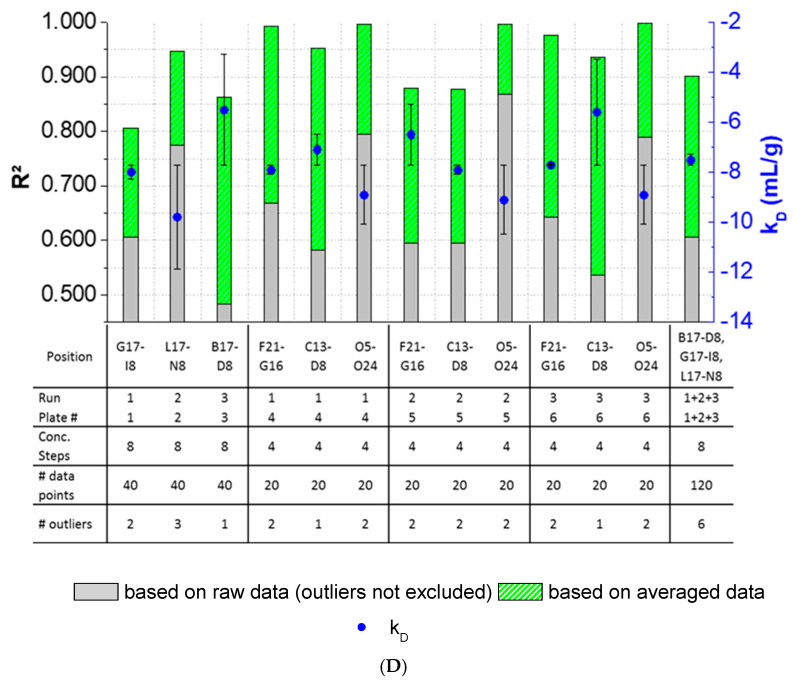
Variations of the coefficients of correlation (*R^2^*) as a function of the excluded outliers and number of data points for: positive *k_D_:* (**A**) LysSig2243 and (**B**) LysSig5161 and negative *k_D_*: (**C**) LysSig2243+NaCl and (**D**) LysSig5161+NaCl. An increase of *R^2^* (based on the excluded outliers) is indicated by stacked green bars. The table contains the plate position, run, and plate number according to Figure 1; Figure 2; number of concentration steps (2, 5, 10, and 14-mg/mL or 2, 3.5, 5, 7, 8, 10, 12, and 14-mg/mL Lysozyme); used data points for the *k_D_* analysis; and number of detected outliers by the Grubbs’ test. Corresponding *k_D_* values are shown as blue spheres on the secondary *y*-axis. Error bars represent the deviation compared to the overall average *k_D_* collected for all runs and plates.

The results showed that outlier test adaption and the exclusion of detected outliers within datasets are straightforward and generally improved the *k_D_* analysis in most cases (Figure 4). Only in two cases (Pos. B17–C12, Figure 4B), the exclusion of one and two outliers, respectively, did not improve the correlation of the curve. In all other cases, the *R^2^* was improved when outliers were excluded for the *k_D_* analysis. The analysis and exclusion of outliers was highly effective for formulations demonstrating negative *k_D_* (Figure 4C,D), since the *R^2^* values were strongly improved by +23.3% (LysSig2243+NaCl) and +42.0% (LysSig5161+NaCl). The *k_D_* calculated with 20 data points showed a *CV* < 5.1%, while an analysis based on 40 data points resulted in a maximum *CV* of 4.5%. The *CV* of a *k_D_* analysis based on 120 data points was < 1.9%, presenting the lowest deviation compared to the 20- and 40-data point analyses. Berg et al. [18] discussed that the evaporation of aqueous solutions in 96-, 384-, and 1536-well plates were found out to be stronger at the corner and edge wells [33] of the plate than in the center wells. Interestingly, the maximum deviation was shown when the *k_D_* analysis included edge wells (results of 20 data points). The edge well samples (A1–A20) showed a higher *CV* (4.6%) compared to the center well samples (L17–M12, *CV* 2.4%). The *CV* obtained with 40 data points and edge wells (A1–B16) was also 37% higher compared to the considered samples at the center wells (e.g., F1–G16). The more data points were used for the *k_D_* analysis, the more negligible the impact of including the edge wells became. As a result, when the *k_D_* analysis was based on 20 data points, four concentration steps and including the edge wells (i.e., row A), the deviation of the *k_D_* was higher compared to more data points and samples placed at the center well positions. However, low amounts of API for analytical characterization are preferable, and thus, the *k_D_* values determined by including less data points with a *CV* < 5% are still acceptable. In summary, the *k_D_* analysis based on four concentration steps (i.e., 20 data points, center wells, 150-mg lysozyme) provided similar results compared to eight concentration steps, whilst benefiting from the reduction of the lysozyme substance (83%) compared to the 120-data points approach (900-mg lysozyme).

### 3.2. k_D_ Measurement as a Function of Plate Position and Elapsed Time

**Figure 5:**
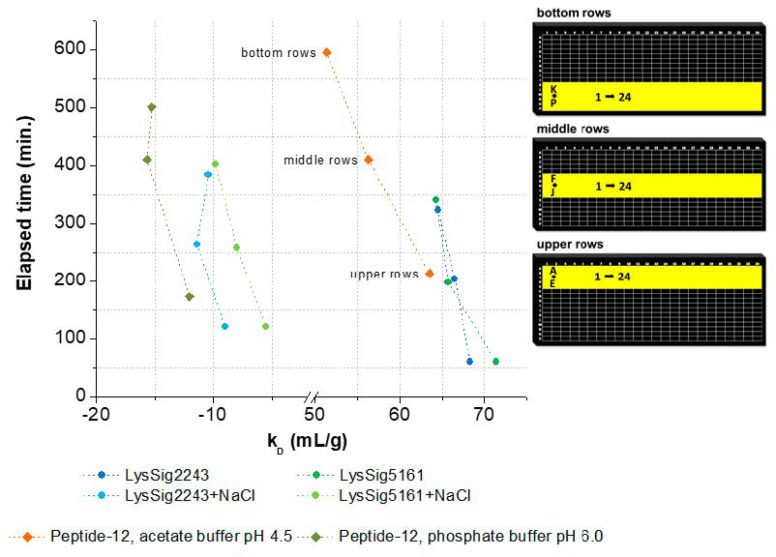
*k_D_* as a function of elapsed time and the corresponding plate positions (bottom, middle, and upper rows). Spheres present *k_D_* collected for lysozyme, and squares show *k_D_* collected for peptide-12 in acetate, pH 4.5 (orange), or phosphate, pH 6.0 (olive green), buffer. *k_D_* was calculated using the wells as highlighted in yellow on the right-hand side. Exact well numbers can be found in Appendix B.

The *k_D_* decreased related to the elapsed time and corresponding plate position (bottom, middle, and upper rows) (Figure 5). In this context, elapsed time is defined as the amount of time that passes from the beginning of the DLS experiment (first well, e.g., upper row: A1) to its end (last well, e.g., upper row: P24). The decrease of *k_D_* was as follows: LysSig2243 −5.9% and LysSig5161 −11.0%, LysSig2243+NaCl −13.5% and LysSig5161+NaCl −43.9%, peptide-12 in acetate buffer, pH 4.5, −19.1%, and peptide-12 in phosphate buffer, pH 6.0, −29.8%. The *k_D_* plots and DLS autocorrelation functions can be found in the Supplementary Materials (Figures S1 and S3 and Tables S1 and S2, respectively). The end time of the data acquisitions was not similar for the plates and formulations, as expected. This discrepancy in the end time was likely due to the automatic laser power attenuation of the plate reader. The recommended working volume of the used microtiter plates is 5–40 µL, as given by the manufacturer. A further recommendation for sufficiently robust DLS experiments is also to ensure that the sample volume in the well will not become lower than the minimum working volume during analysis. However, due to the long residence time of the plate in the plate reader at 25 °C, it is very likely that the initial sample volume of 30 µL per well in the bottom rows was reduced over time.

**Figure 6:**
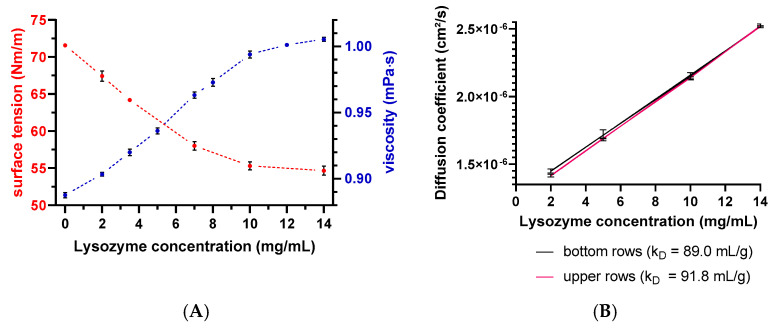
(**A**) Surface tension and dynamic viscosity data as a function of the lysozyme concentration. Red spheres present the surface tension determined by the Wilhelmy plate method, and viscosity data are shown as blue spheres on the secondary *y*-axis. Error bars represent the standard deviation of 4 measurements. Error bars are possibly smaller than the symbols. (**B**) Diffusion coefficient as a function of the lysozyme concentration and plate position. The red line represents *D* collected in upper rows, and the black line represents *D* collected in the bottom rows. Error bars represent the standard deviations of 6 measurements for the diffusion coefficients by DLS.

Evaporation of the solvent can negatively affect the analysis through concentrating effects (e.g., increasing the protein concentration and ionic strengths). Solvent evaporation led to an increase in lysozyme or peptide-12 concentration and, thus, to altered *D* and, consequently, *k_D_* values. The effect was already noticeable for all samples in the middle rows and elapsed times > 150 min indicated by a deviation of the expected linear relationship (Figure 5). The precise concentration at the time of measurement was unknown, since changes in concentration after the start of data acquisition could not be directly monitored. Furthermore, the size and geometric characteristics of the wells can contribute to an increase of the surface effects and, thus, to accelerated evaporation. Lysozyme is positively charged at a solution pH of 4.5 due to the isoelectric point of 11.4. These charged molecules create an electric field in the solution close to the liquid–air interface. Furthermore, intermolecular interactions are mediated by several forces and interactions (e.g., electrostatic forces, hydration shell, and adhesion between molecules and the polystyrene surface of the well) [34,35]. Capillary forces could be also responsible for the enhanced evaporation due to an increased meniscus curvature [18] and capillary flows. The first steps of evaporation can be explained by a competition between the surface tension gradients (e.g., Marangoni flows) and capillary flows. Furthermore, Marangoni flows caused by the presence of amphiphilic molecules (e.g., proteins) can force the fluid to circulate inwards in the other direction of the capillary flows [34,36] (i.e., dissolved lysozyme might compensate the capillary flows related to evaporation). The surface tension of lysozyme decreased with the increasing lysozyme concentration (Figure 6A), which is in good agreement with the literature [37]. The measured diffusion coefficients of the low concentrated samples increased (2-mg/mL lysozyme in the absence of NaCl: 2.5% and in the presence of 400-mM NaCl: 2.8%), dependent on the plate position (upper vs. bottom rows) and the elapsed time (Figure 6B). Accordingly, the solvent evaporation during the experiment led to an increase in lysozyme concentration, as well as solution viscosity and, thus, to increased *D*. However, the magnitude of the supposed changes in the concentration and viscosity cannot be considered and adapted during the DLS experiment. Higher concentrated samples (e.g., 10 or 14-mg/mL lysozyme) were possibly less prone to evaporation (see Figure 6A, e.g., due to a higher solution viscosity and stronger intermolecular interactions) over the elapsed time and showed a less pronounced increase of *D* (Figure 6B: 0.60% and 0.04%, respectively). At a higher lysozyme concentration, more water molecules were bound to the surfaces of lysozyme molecules (i.e., formation of a hydration shell), and thus, the total number of freely movable water molecules was reduced. Furthermore, the higher initial sample viscosity reduced the mobility of the water molecules, resulting in a reduced evaporation tendency [38]. The obtained linear function (*D* vs. *c*) was shifted towards decreased *k_D_* values (Figure 6B, upper rows: *k_D_* = 91.8 mL/g and bottom rows: *k_D_* = 89.0 mL/g). Furthermore, the solvent evaporation can also alter the ionic strength of the solution, which is of particular interest for low-salt concentration samples, since small changes in the salt concentration will have an impact on the intermolecular interactions and, thus, the *k_D_* [39].

### 3.3. Intra-Plate Variability of Plate-Based DLS Measurements

The intra-plate variation was determined for the goal of detecting patterns, such as edge effects or trends along rows or columns, and to assess the variability of the obtained results between positions.

**Figure 7:**
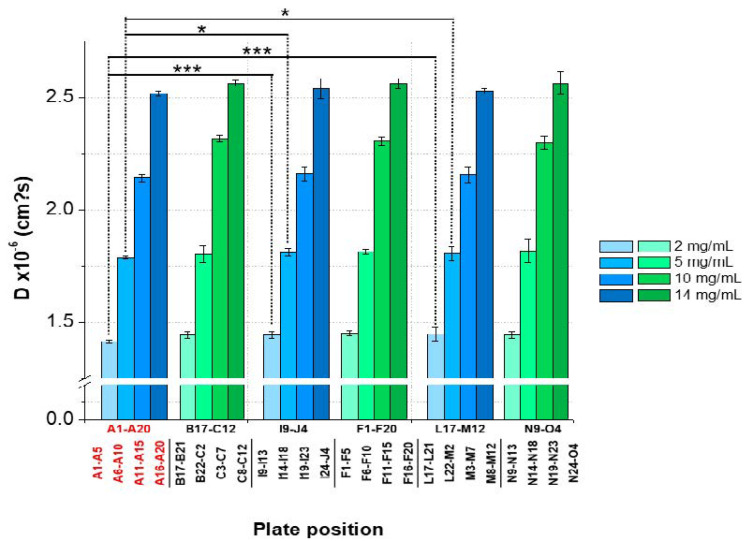
Statistical evaluation (intra-plate coefficient of variation (*CV*)) with regard to the diffusion coefficients as a function of plate position collected for two lysozyme batches (LysSig2243: blue and LysSig5161: green); 4 concentrations (2, 5, 10, and 14-mg/mL lysozyme); and 5 replicates. Edge rows (A1–A20) are marked in red. The plate design is shown in Figure 1. Significant differences are indicated by asterisks: * *p* < 0.05 and *** *p* < 0.001.

**Figure 8:**
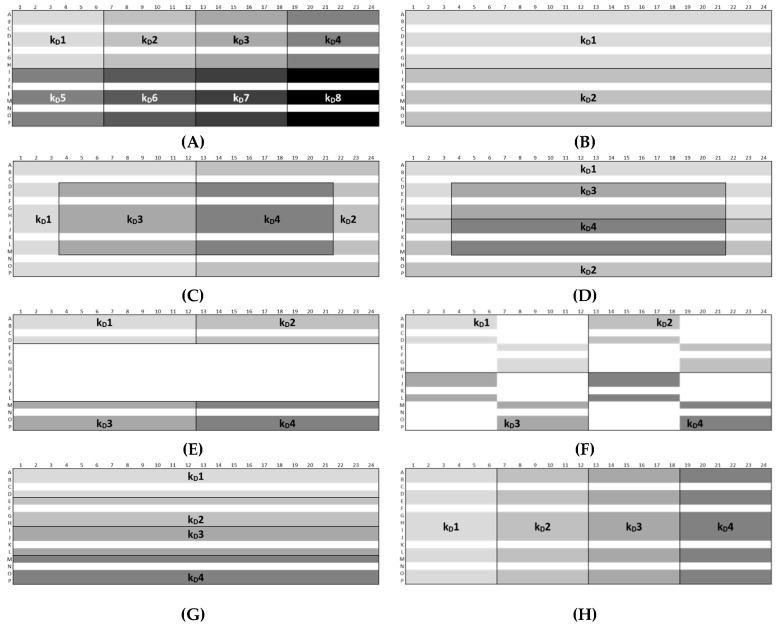
Plate designs (**A**)–(**H**) of the approaches Ac and Ph. “*k_D_#*” shows the wells used for the calculation of the single *k_D_* values. The white area shows omitted wells.

The results showed an overall *CV* < 2% between the plate positions for samples with low, medium, and high lysozyme concentrations for both batches. However, significant variations between the plate positions (A1–A5 and A6–A10) concerning 2 and 5-mg/mL LysSig2243 formulations were observed (Figure 7). The samples placed at the edge wells A11–A15 and A16–A20 (10 and 14-mg/mL LysSig2243, respectively) showed *CV* of 1.8% and 1.5% of the diffusion coefficients, respectively. However, the differences were not statistically significant (*p* > 0.05). The variance analysis of the LysSig5161 formulations showed similar results. There was no significant difference between the center positions: B17–C12, F1–F20, L17–M12, or N9–O4. This study indicated that the arrangement of samples in the edge wells and lower rows led to a significant increase of variations impeding a reliable determination of *D*. The plate design for peptide-12 was adapted based on previous lysozyme variation studies. The peptide-12 concentration series (2, 4, 6, 8, and 10 mg/mL) for the intra-plate variability study was positioned as six replicates of each concentration vertically instead of horizontally. The evaluation of *D* showed that the adapted plate design with vertical sample replicates for peptide-12 led to the improved precision of *D*. The results were based on various geometric sample matrices of *k_D_* calculations and selected with respect to studying the variabilities between (Table 1, Figure 8): (i) edge vs. center plate positions, (ii) horizontal vs. vertical vs. diagonal sample layouts, and (iii) upper and bottom row samples.

For peptide-12 in acetate buffer, pH 4.5, approach Ac-C showed the lowest intra-plate *CV* (1.6%). Here, the sample replicates to determine the *k_D_1* and *k_D_2* were arranged in edge rows (A and P) and columns (1 and 24), and the calculations were based on 12 replicates each of 2, 4, 8, and 10 mg/mL and 16 replicates of 6-mg/mL peptide-12 samples (i.e., 64 “# data points (single *k_D_*)”). The *k_D_3* and *k_D_4* were solely calculated by sample replicates arranged in the center wells and 12 replicates of 2, 4, 8, and 10 mg/mL and eight replicates of 6-mg/mL peptide-12 samples (i.e., 56 “# data points (single *k_D_*)”). The lowest and highest concentrations of peptide-12 were placed in the edge columns 1 and 24; thus, the curve progression was less susceptible to fluctuations regarding the diffusion coefficients. As a result, the four single *k_D_* varied only slightly (*SD* ±0.9 mL/g). Approach Ac-E showed the highest variability between the *k_D_*, since the majority of the sample replicates were arranged in edge wells (i.e., rows: A and P and columns: 1 and 24). The *k_D_* analysis included only a small number of data points (i.e., 30; *CV* 14.1%, *SD* ±8.1 mL/g), and minor deviations of *D* became increasingly relevant, affecting the calculated *k_D_* values. Approach Ac-B showed a low variability (*CV* 6.8%, *SD* ±3.8 mL/g), since the single *k_D_* analysis was based on 120 data points (i.e., 24 replicates at five concentrations). However, the *k_D_* obtained from 30 data points (approaches 1 and 6) showed also a *CV* < 10%. The samples of approach Ac-G were horizontally ordered, and the single *k_D_* analysis was based on 60 data points (*CV* 9.2%, *SD* ±5.2 mL/g). A moderate variability was expected for approach Ac-H, since the sample replicates (60 data points) were vertically placed on the plate, providing equal elapsed times for each single *k_D_*. However, the *CV* was 9.4%, and the *SD* of ±5.2 mL/g also indicated that the intended compensating effects were not relevant. The total span of all 34 *k_D_* collected by eight approaches was 39.2% (*k_D low_*: 47.4 mL/g and *k_D high_*: 65.9 mL/g). This suggests that optimized plate designs have an impact on the quality of obtained *k_D_*. For peptide-12 in phosphate buffer, pH 6.0, approach Ph-B was also expected to show a low variability, since the single *k_D_* analysis was based on 120 data points. The *CV* (4.0%) and *SD* (±0.6 mL/g) were the lowest within the eight approaches, and the span between the four single *k_D_* was also quite low (5.4%). Approach Ph-E showed again the highest *CV* (20.4%) and *SD* (±2.9 mL/g) of the *k_D_*, since the sample replicates were arranged mainly in edge wells and based on 30 data points. The *k_D_* obtained from 30 data points (approaches Ph-A and Ph-F) presented *CV* > 10% and high spans of *k_D_* (37.6% and 22.2%, respectively). Approaches Ph-C, Ph-D, Ph-G, and Ph-H also showed a *CV* > 10%. The total span of all 34 *k_D_* collected by the eight approaches was 37.6% (*k_D low_*: −14.1 mL/g and *k_D high_*: −15.8 mL/g) and was in a similar range compared to peptide-12 in acetate buffer, pH 4.5. The calculation of reliable and precise *k_D_* for peptide-12 was dependent on the position of the sample replicates on the plate, as well as the value of the *k_D_*. Overall, the results indicated that the plate designs according to approaches Ac-B, Ph-B or Ac-C, Ph-C provided a reliable *k_D_* analysis presenting very low *CV*, whereas approaches Ac-E, Ph-E showed the inferior values from the view of the *SD*, *CV*, and “span *k_D_*”. High positive *k_D_* values showed a lower variability compared to low and negative *k_D_* values. However, it was also indicated that using more data points for the *k_D_* analysis did not necessarily result in a superior variability (120 vs. 60 vs. 30 data points). The *k_D_* analysis exclusively based on center wells showed lower *SD* and *CV* (<2%) compared to edge wells (5–12%) or a combination of edge and center wells (2–5%). It was also pointed out that five replicates of each concentration were adequate for a reliable determination of the diffusion coefficients and *k_D_*, which was confirmed by the low *SD* and *CV*, respectively.

### 3.4. Inter-Plate Variability of Plate-Based DLS Measurements

The inter-plate variability refers to variations between runs of sample replicates on different plates or runs (i.e., plate-to-plate/run-to-run consistencies). Samples of high (14 mg/mL), medium (5 and 10 mg/mL), and low (2 mg/mL) lysozyme concentrations were used to calculate the inter-plate *CV*.

**Figure 9:**
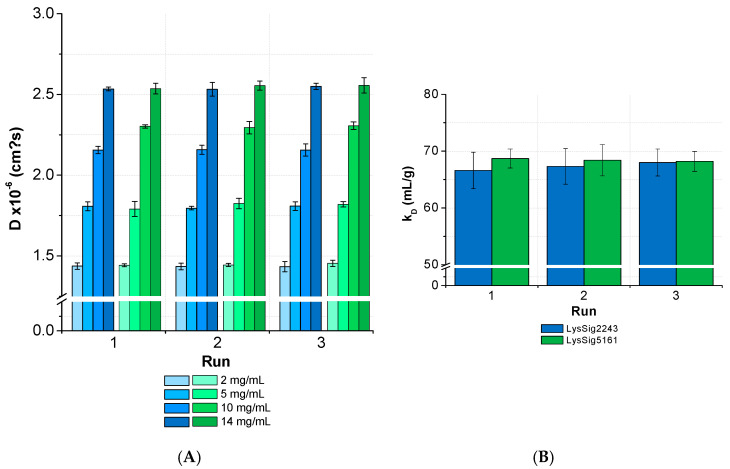
Statistical evaluation (inter-plate *CV*) with regards to (**A**) diffusion coefficients collected for two lysozyme batches (LysSig2243: blue and LysSig5161: green), 4 concentrations, 5 replicates, and 3 runs. The plate design is shown in Figure 2. (**B**) The *k_D_* obtained from the *D* of 4 concentrations (2, 5, 10, and 14-mg/mL lysozyme) and 5 replicates at 3 plate positions collected in 3 separate runs (9 total *k_D_* of each batch).

The ANOVA revealed no significant differences (*p* < 0.05) between the *D* and *k_D_* collected from several runs and by separately analyzed plates for lysozyme (Figure 9). The overall *CV* was < 3% for the samples with low, medium, and high lysozyme concentrations (LysSig2243 and LysSig5161). The ANOVA of the formulations containing sodium chloride provided similar results and, thus, a significant run-to-run consistency. The average *CV* of the diffusion coefficients for samples with low, medium, and high lysozyme concentrations were 3.2% (LysSig2243+NaCl) and 2.9% (LysSig5161+NaCl). The highest *CV* between different concentrations were 3.9% and 3.5% (LysSig2243+NaCl and LysSig5161+NaCl, respectively). The ANOVA also revealed no significant differences between the runs.

**Figure 10:**
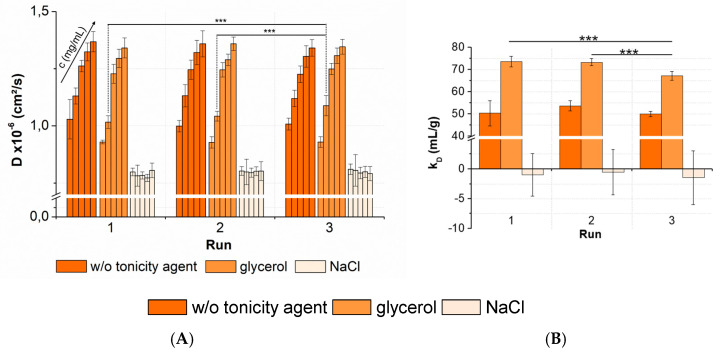
Statistical evaluation (inter-plate *CV*) with regards to (**A**) diffusion coefficients collected for three peptide-12 formulations in acetate buffer, pH 4.5, in the absence or presence of tonicity agents (w/o tonicity agent: dark orange, glycerol: orange, NaCl: slight orange) concentrations (in ascending order: 2, 4, 6, 8, and 10-mg/mL peptide-12); 5 replicates; and 3 runs. Significant differences are indicated by asterisks: *** *p* < 0.001. The plate design is shown in Appendix A, Figure A2. (**B**) The *k_D_* obtained from the *D* of 5 concentrations (2, 4, 6, 8, and 10-mg/mL peptide-12) and 5 replicates at 3 plate positions collected in 3 separate runs (9 total *k_D_* of each formulation).

For this study, the selected formulations included peptide-12 formulated in acetate buffer, pH 4.5, in combination with glycerol or sodium chloride as the tonicity agent (Figure 10). The plate was measured by DLS on three consecutive runs. Samples that were arranged solely at the center wells and edge wells were omitted.

For peptide-12 formulated in acetate buffer, pH 4.5, the results showed a *CV* < 4% for the collected *D*. The averaged *k_D_* of peptide-12 in acetate buffer, pH 4.5, in the absence of a tonicity agent was 51.3 ± 3.6 mL/g (*CV* 7.0%). The ANOVA revealed no significant variations between runs, as well as between the *k_D_* collected in several runs. A statistical evaluation was also performed for the peptide-12 formulations combined with a nonionic (glycerol) and ionic (sodium chloride) tonicity agent. The results showed *CV* < 5% for samples of peptide-12 in the presence of glycerol. The ANOVA concluded no significant variations between runs. An exception was the formulation at 4-mg/mL peptide-12 in the presence of glycerol. A post-hoc *t*-test indicated that run 3 was significant different compared to runs 1 and 2. The runs were performed consecutively, and the samples were stored at +5 °C, minimizing the potential degradation processes. The DLS raw data showed a second particle population in run 3 (*R_H_* = 42.8 ± 7.9 nm, *I_Peak2_* = 11.6%). Larger particles move at lower speeds than smaller particles, and thus, the diffusion coefficients were affected by the presence of larger oligomers or soluble aggregates in the sample. Additionally, the *k_D_* of the formulations in the presence of glycerol showed a significant difference between the three runs (*p* = 0.0003). The averaged *k_D_* was 71.4 ± 3.6 mL/g (*CV* 5.0%), and run 3 was significantly different compared to run 1 and run 2. As previously discussed, diffusion coefficients of 4-mg/mL peptide-12 formulations were significantly different due to a second particle population observed in the DLS experiment. This small number of larger particles affected the reliability of the *k_D_* calculations based on *D*. If run 3 is excluded from the dataset, the averaged *k_D_* is 73.4 mL/g, showing a quite low *SD* (±1.8 mL/g) and *CV* (2.5%). In the presence of sodium chloride, peptide-12 showed a negative *k_D_* and an insufficient correlation between the diffusion coefficients and peptide concentrations (*R^2^* << 0.4). The results showed an average *CV* of 4% for *D* and an average *k_D_* of −1.0 mL/g, with a high *SD* (±3.5 mL/g) and *CV* (357%). The slope of the linear relationship of diffusion and concentration (Equation (2)) approached zero, and the variance of the individual diffusion coefficients increased. Minor changes in the diffusion coefficients within the sample replicates led to a strong fluctuation and poor linear correlation of the data points. It was assumed that the properties and composition of a liquid formulation affected the precision and reliability of the *k_D_* analysis. The trend, that high *k_D_* values showed lower *CV* than low *k_D_* values, was associated with the value of the *k_D_*. However, it was pointed out that five replicates of each concentration were adequate for a reliable determination of the *D*, as well as *k_D_*, which was confirmed by a low *SD* and *CV* < 10%.

### 3.5. Plate Design Recommendations for High-Throughput Formulation Screening

The recommendations were defined in order to achieve mature plate designs, as well as reliable *k_D_* analyses (e.g., low *SD* and *CV*), and were based on the findings of our study. The API availability during early formulation development is usually limited. Some material is needed for the initial studies, such as solubility testing (~70 mg) and chemical stability studies (~15 mg). Realistically, 100–200-mg API may be available for plate-based formulation screenings. For instance, 370 mg of peptide-12 was used for the inter-plate study tested within this work, visualizing that the material consumption was much higher than the generally available amount of API.

**Figure 11:**
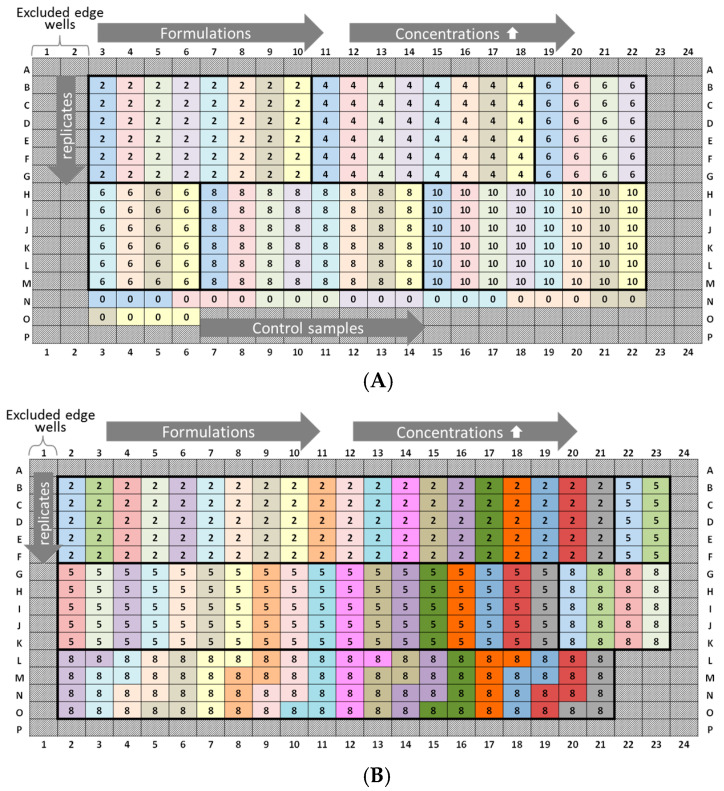
Plate design recommendations for *k_D_* determination and the screening of prototype formulations. (**A**) “Low risk”: 8 formulations (marked by blue to yellow), based on a horizontally arranged concentration series (2, 4, 6, 8, and 10-mg/mL API), and 6 replicates of each concentration are positioned vertically (=240 sample wells, elapsed time (well B3 → M22): 5 h). Control samples are optionally placed at the end positions. Total net amount API: 44 mg. (**B**) “High quantity”: 20 formulations (marked by blue to gray), based on a horizontally arranged concentration series (2, 5, and 10-mg/mL API), and 5 replicates of each concentration are positioned vertically (=300 sample wells, elapsed time (well B2 → O21): 6.25 h). Placebo samples are not placed on the microtiter plate. Total net amount API: 50 mg.

Inter-plate variability studies are time- and material-consuming and, thus, difficult to include in general predevelopment studies of drug candidates. Therefore, the following two plate design recommendations (Figure 11) were optimized, using as little of the API as possible without a loss of data integrity. The exclusion of edge wells (i.e., rows A and P (2 × 24 wells) and columns 1 and 24 (2 × 16 wells)) is highly recommended, and our study revealed a reliable *k_D_* analysis based on four API concentration steps instead of 6–10 [6,40,41,42,43]. However, the lowest and highest concentrations should be considered carefully, while intermediate concentrations may be omitted in order to save the API. Our results suggested an association between the time elapsed and *k_D_* values. A vertical sample arrangement of five replicates is preferred, since an improved precision of the diffusion coefficients was achieved. Low concentration samples should be positioned on upper rows to minimize the effect of evaporation during data acquisition. Edge well (i.e., rows A and P (2 × 24 wells) can be optionally filled with liquid (e.g., water or buffer) for additional thermal insulation and, thus, for reducing the effect of evaporation of the neighboring sample wells. An outlier test should be applied to detect and exclude outliers within the datasets, since the curve progression can be affected positively (*R^2^* > 0.9).

## 4. Conclusions

High-throughput methods may suffer increased variances of data. In this work, we qualified a DLS plate reader method in a 384-well plate format for the *k_D_* analysis. Our intra-plate study indicated that samples placed in edge wells and bottom rows led to a significant variation in the *k_D_* analysis. The *k_D_* as a function of elapsed time showed that solvent evaporation during measurements can lead to increased protein concentrations and, thus, to altered *k_D_*. Therefore, concentration series of prototype formulations have to be ordered alternately on the plate for direct head-to-head comparisons. The ANOVA revealed no significant differences (*p* < 0.05) between three independent runs (inter-plate variability). However, peptide-12 in the presence of glycerol showed a significant difference in run 3, due to larger intermediately formed particles disturbing the calculation of the *k_D_*. The appearance of formed particle species between formulation preparation and analysis highlighted the importance of sterile filtration (0.22 µm or 0.1 µm) prior to light scattering measurements. Interestingly, the *k_D_* analyses based on 120 data points showed only minor differences and variability compared to 20 or 30 data point approaches. However, a major advantage of less data point approaches is the lower amount of API required. Outlier tests to detect and exclude outliers, leading to an improved curve fitting (i.e., a higher coefficient of correlation (*R^2^*)) should also be considered and implemented in plate-based formulation screening studies. The determination of reliable *k_D_* values was also dependent on the extent of the *k_D_* value and sample position. High positive *k_D_* values and *k_D_* analyses exclusively based on center wells showed a lower variability compared to negative *k_D_* values and replicates placed in edge wells. The exclusion of edge wells is therefore highly recommended. Furthermore, the *k_D_* analysis should be based on four well-considered concentration steps, whereby intermediate concentrations may be omitted to save API material. A vertical arrangement of the samples, including five replicates (considering the exclusion of one possible outlier), is also preferred. Low concentrations should be positioned on upper rows, minimizing the effect of concentration-dependent solvent evaporation. Furthermore, edge wells can be optionally filled with liquid, exploiting the thermal insulation effect on sample evaporation. Finally, we presented refined plate designs based on statistically evaluated data for the high-throughput screening of eight to 20 formulation compositions with less than 50-mg API.

## Figures and Tables

**Table 1:** Comparison between approaches to calculate the diffusion self-interaction parameter (*k_D_*) of peptide-12 in (Approach Ac) acetate buffer, pH 4.5, and (Approach Ph) in phosphate buffer, pH 6.0. In order to ensure comparability of the *k_D_*, the diffusion coefficients were submitted to a Grubbs’ test. Detected outliers were excluded from the dataset and not used for the *k_D_* calculations. The average intra-plate coefficient of variation (*CV*) was obtained by averaging the individual *CV*. The total averaged *k_D_*, including 34 *k_D_* obtained by 8 approaches (A–H), is shown in the right column. “# data points (single *k_D_*)” describes the number of sample replicates with regards to each concentration for the single *k_D_* determination (e.g., 6 replicates at 5 concentrations = 30 data points). “Span *k_D_*” is defined as percentage difference between the considered *k_D_* and total averaged *k_D_*.

**Approach Ac**									
	A	B	C	D	E	F	G	H	Av.
**Average *k_D_* (mL/g)**	59.8	56.3	55.6	57.4	57.1	54.7	56.4	55.7	57.0
***SD*** ***k_D_* (mL/g)**	5.4	3.8	0.9	6.0	8.1	4.8	5.2	5.2	5.1
**# single *k_D_***	8	2	4	4	4	4	4	4	34
**# data points (single *k_D_*)**	30	120	64/56	64/56	30	30	60	60	n.a.
***CV*** **(%)**	9.0	6.8	1.6	10.4	14.1	8.7	9.2	9.4	8.9
**Span *k_D_* (%)**	25.8	9.1	3.7	21.0	24.5	17.9	16.9	20.6	39.2
**Approach Ph**									
	A	B	C	D	E	F	G	H	Av.
**Average *k_D_* (mL/g)**	−14.8	−15.6	−14.5	−14.7	−14.1	−15.8	−14.7	−14.5	−14.8
***SD k_D_*** **(mL/g)**	2.5	0.6	2.0	2.3	2.9	1.6	2.0	1.5	2.0
**# single *k_D_***	8	2	4	4	4	4	4	4	34
**# data points (single *k_D_*)**	30	120	64/56	64/56	30	30	60	60	n.a.
***CV* (%)**	16.8	4.0	13.5	15.6	20.4	10.2	13.7	10.6	13.4
**Span *k_D_* (%)**	37.6	5.4	26.7	30.8	33.2	22.2	26.7	19.7	37.6

## Data Availability

No new data were created or analyzed in this study.

## Supplementary Materials

The following are available online at https://www.mdpi.com/1999-4923/13/2/172/s1: Figure S1. Diffusion coefficient as a function of the lysozyme concentration formulated in 20-mM acetate buffer, pH 4.5, in the absence or presence of 400-mM NaCl and the plate position. Figure S2. Diffusion coefficient as a function of the peptide-12 concentration formulated in 30-mM buffers in the absence of tonicity agents and the plate position. Figure S3. Diffusion coefficient as a function of the lysozyme concentration formulated in 20-mM acetate buffer, pH 4.5 (for two lysozyme batches LysSig2243: blue and LysSig5161: green), and the run. Figure S4. Diffusion coefficient as a function of the peptide-12 concentration formulated in 30-mM buffers in the absence or presence of tonicity agents (w/o tonicity agent: dark orange, glycerol: orange, and NaCl: slight orange) and the run. Table S1. DLS autocorrelation functions (ACF) for the *k_D_* analysis for lysozyme in 20-mM acetate buffer, pH 4.5, in the absence and presence of 400-mM NaCl. Table S2. DLS autocorrelation functions (ACF) for the *k_D_* analysis for peptide-12 in 30-mM acetate buffer, pH 4.5, or phosphate buffer, pH 6.0, in the absence and presence of tonicity agents (glycerol and NaCl).

## Appendix B

Supplementary information to Figure 5: well numbers for upper rows: wells A1–B16 for LysSig2243 and LysSig5161, wells B17–D8 for LysSig2243+NaCl and LysSig5161+NaCl, and wells A1–24+B1–24+D1–24+E1–24 for peptide-12 in acetate, pH 4.5, and phosphate, pH 6.0, buffer; for middle rows: wells F1-G16 for LysSig2243 and LysSig5161, wells G17–I8 for LysSig2243+NaCl and LysSig5161+NaCl, and wells G1–24+H1–24+I1-24+J1–24 for peptide-12 in acetate, pH 4.5, and phosphate, pH 6.0, buffer; and for bottom rows: wells K1–L16 for LysSig2243 and LysSig5161, wells L17–N8 for LysSig2243+NaCl and LysSig5161+NaCl, and wells L1–24+M1–24+O1–24+P1–24 for peptide-12 in acetate, pH 4.5, and phosphate, pH 6.0, buffer.
