# Supplementary material for: Microwell Plate-Based Dynamic Light Scattering as a High-Throughput Characterization Tool in Biopharmaceutical Development
Source: Pharmaceutics. 2021 Jan 27;13(2):172. doi: 10.3390/pharmaceutics13020172 (PMC7911513; doi:10.3390/pharmaceutics13020172)
Supplement: Supplementary file 1 [file pharmaceutics-13-00172-s001.pdf]

pharmaceutics

Supplementary Materials

Microwell Plate-Based Dynamic Light Scattering as a High-Throughput Characterization Tool in Biopharmaceutical Development

Katharina Dauer, Stefania Pfeiffer-Marek, Walter Kamm and Karl G. Wagner

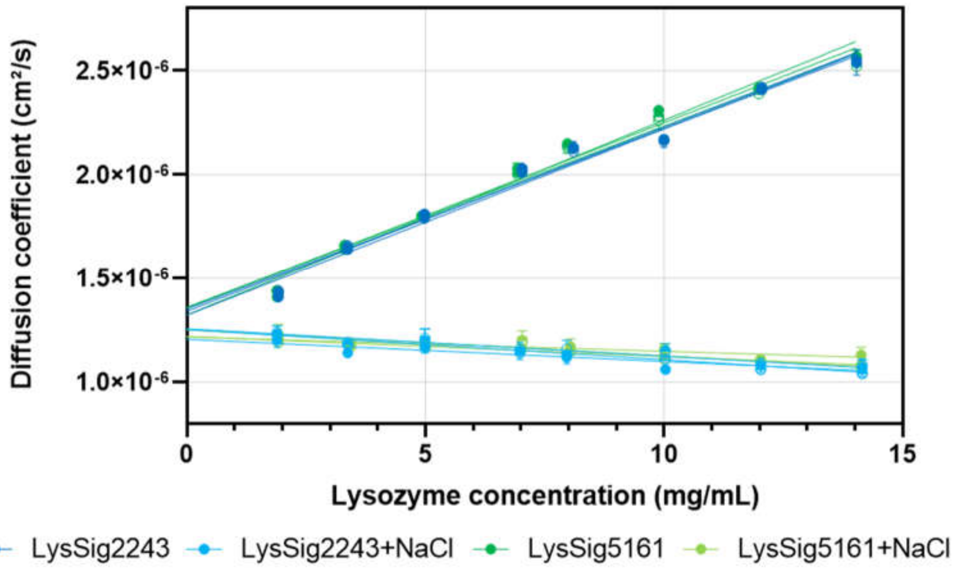

Figure S1. Diffusion coefficient as a function of lysozyme concentration formulated in 20 mM acetate buffer pH 4.5 in absence or presence of 400 mM NaCl. Filled circles represent D collected in upper rows, half-filled circles represent D collected in middle rows, and empty circles represent D collected in bottom rows. The y-intercept provides concentration-independent D_0. Error bars represent the standard deviation of 6 measurements for the diffusion coefficients by DLS.

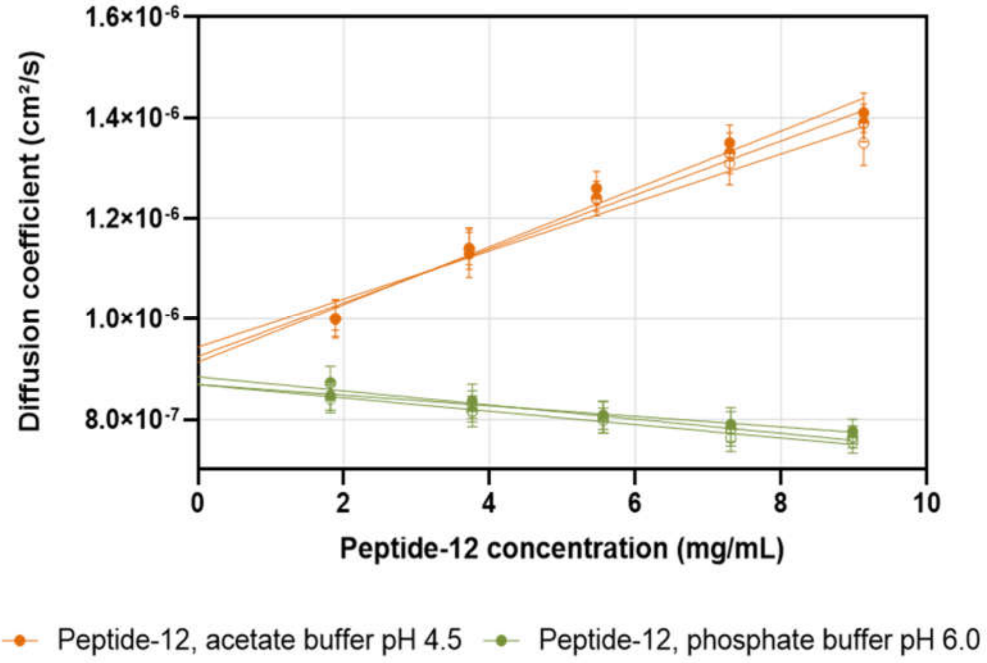

Figure S2. Diffusion coefficient as a function of peptide-12 concentration formulated in 30 mM buffers in absence of tonicity agents. Filled circles represent *D* collected in upper rows, half-filled circles represent *D* collected in middle rows, and empty circles represent *D* collected in bottom rows. The y-intercept provides concentration-independent D_0. Error bars represent the standard deviation of 6 measurements for the diffusion coefficients by DLS.

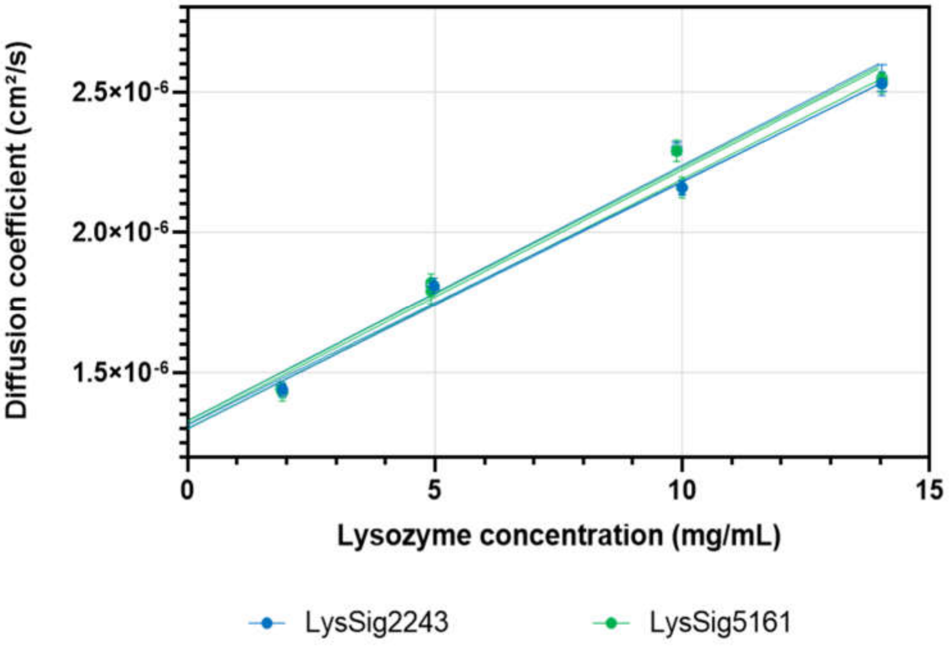

Figure S3. Diffusion coefficient as a function of lysozyme concentration formulated in 20 mM acetate buffer pH 4.5 (for two lysozyme batches LysSig2243: blue, LysSig5161: green). Filled circles represent *D* collected in run 1, half-filled circles represent *D* collected in run 2, and empty circles represent *D* collected in run 3. The y-intercept provides concentration-independent D_0. Error bars represent the standard deviation of 6 measurements for the diffusion coefficients by DLS.

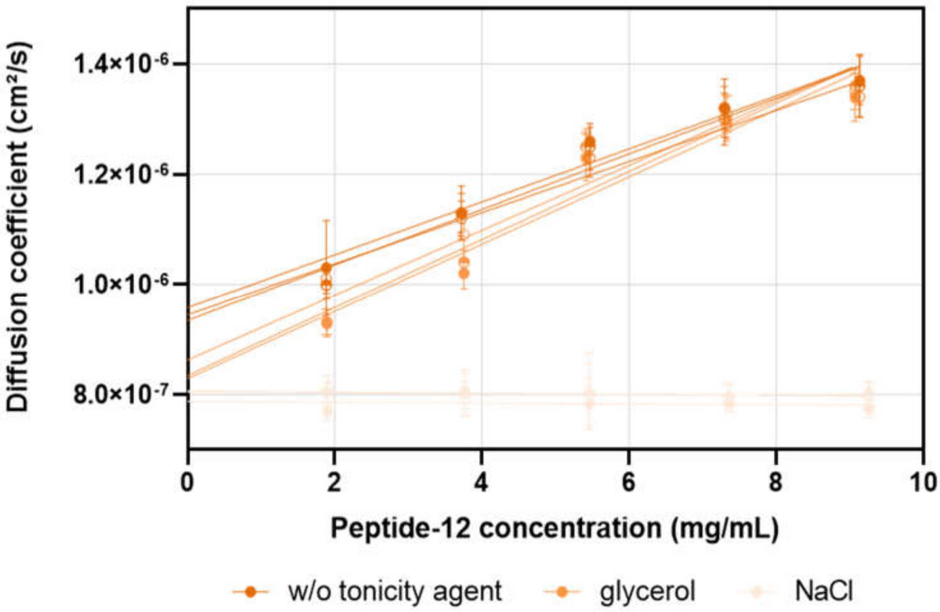

Figure S4. Diffusion coefficient as a function of peptide-12 concentration formulated in 30 mM buffers in absence or presence of tonicity agents (w/o tonicity agent: dark orange, glycerol: orange, NaCl: slight orange). Filled circles represent *D* collected in run 1, half-filled circles represent *D* collected in run 2, and empty circles represent *D* collected in run 3. The y-intercept provides concentration-independent D_0. Error bars represent the standard deviation of 6 measurements for the diffusion coefficients by DLS.

Table S1. DLS autocorrelation functions (ACF) for k_D analysis for lysozyme at 10 mg/mL concentration in 20 mM acetate buffer pH 4.5 in absence and presence of 400 mM NaCl. SOS (sum-of-squares) and baseline are parameters for judging the "goodness" of the autocorrelation curves. Composition code is given on left hand side

Formulation	Baseline	SOS	ACF
LysSig2243 AcBuffer_pH4.5_none	1.000 ± 0.000	0.394 ± 0.327	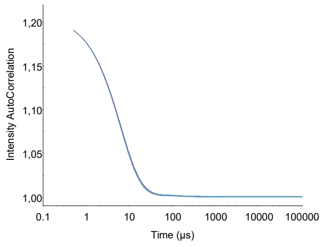
LysSig2243 AcBuffer_pH4.5_NaCl	1.000 ± 0.000	0.097 ± 0.102	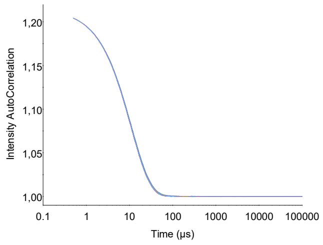

LysSig5161 AcBuffer_pH4.5_none	1.000 ± 0.000	0.310 ± 0.245	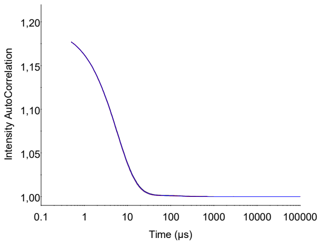
LysSig5161 AcBuffer_pH4.5_NaCl	1.000 ± 0.000	0.087 ± 0.083	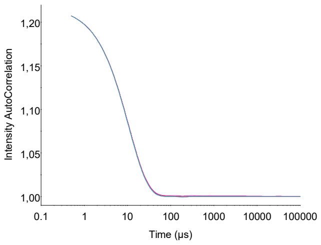

Table S2. DLS autocorrelation functions (ACF) for k_D analysis for peptide-12 in 10 mg/mL concentration in 30 mM acetate buffer pH 4.5 or phosphate buffer pH 6.0 in absence and presence of tonicity agents (glycerol, NaCl). SOS (sum-of-squares) and baseline are parameters for judging the "goodness" of the autocorrelation curves. Composition code is given on left hand side.

Formulation	Baseline	SOS	ACF
Peptide-12 Ac-Buffer_pH4.5_none	1.002 ± 0.007	2.256 ± 3.675	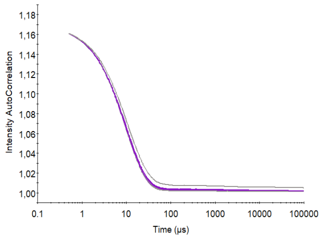
Peptide-12 Ac-Buffer_pH4.5_glycerol	1.003 ± 0.003	7.537 ± 8.399	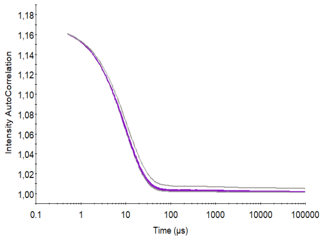
Peptide-12 Ac-Buffer_pH4.5_NaCl	1.002 ± 0.001	2.949 ± 6.004	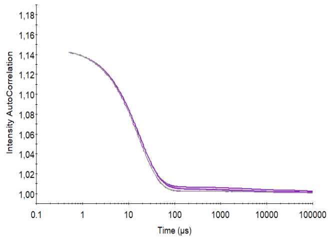
Peptide-12 Phos-Buffer_pH6.0_none	1.001 ± 0.002	1.605 ± 1.739	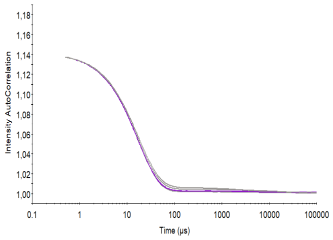

Peptide-12 Phos-Buffer_pH6.0_ glycerol	1.002 ± 0.007	0.647 ± 0.694	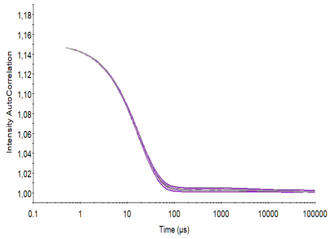
Peptide-12 Phos-Buffer_pH6.0_NaCl	1.002 ± 0.004	1.421 ± 1.505	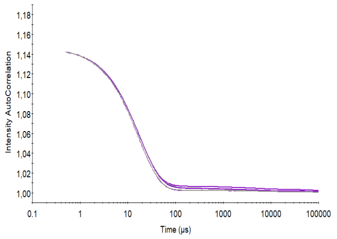
